# A multi-level model for analyzing whole genome sequencing family data with longitudinal traits

**DOI:** 10.1186/1753-6561-8-S1-S86

**Published:** 2014-06-17

**Authors:** Taoye Chen, Patchara Santawisook, Zheyang Wu

**Affiliations:** 1 Department of Mathematical Sciences, Worcester Polytechnic Institute, 100 Institute Road, Worcester, MA 01609-2280, USA

## Abstract

Compared with microarray-based genotyping, next-generation whole genome sequencing (WGS) studies have the strength to provide greater information for the identification of rare variants, which likely account for a significant portion of missing heritability of common human diseases. In WGS, family-based studies are important because they are likely enriched for rare disease variants that segregate with the disease in relatives. We propose a multilevel model to detect disease variants using family-based WGS data with longitudinal measures. This model incorporates the correlation structure from family pedigrees and that from repeated measures. The iterative generalized least squares algorithm was applied to estimation of parameters and test of associations. The model was applied to the data of Genetic Analysis Workshop 18 and compared with existing linear mixed-effect models. The multilevel model shows higher power at practical *p-*value levels and a better type I error control than linear mixed-effect model. Both multilevel and linear mixed-effect models, which use the longitudinal repeated information, have higher power than the methods that only use data collected at one time point.

## Background

Whole genome sequencing (WGS) provides comprehensive collection of genetic variations and thus is promising in discovering novel inheritable factors for both Mendelian and complex traits. Two data properties distinguish WGS from microarray-based genome-wide association study (GWAS). First, WGS data contain rare causal mutations that could have large allelic effect. However, the statistical association for such rare variants is weak at population level because of small allele frequency [1]; therefore, population-based case-control study, which is commonly applied in GWAS, is less powerful for WGS. Second, family design is attractive and commonly applied in WGS studies. Causal rare variants are likely enriched through cotransmission in families. Moreover, pedigree structures allow statistical imputation of genotypes without experimental cost [2]. Additionally, family-based data analyses automatically control for population stratification and are potentially able to incorporate helpful genetic information on phase, effects of parental origin, cotransmission of variants, and so on[3].

Detection of disease variants can also be facilitated by trajectory information on individual changes over time. Longitudinal genetic studies enable a close investigation of both genetic factors that lead to a disease and environmental determinants that modulate the subsequent progression of the disease. In WGS, it is important to develop powerful methods that accommodate both within-family correlation structure and correlation among repeated measures. Here we extend a multilevel model [4,5] to WGS longitudinal family data, which simultaneously accounts for familial and time-series correlations. The implementation is based on the iterative generalized least squares (IGLS) algorithm [6,7], which allows conclusions to be drawn about both genetic and environmental effects while controlling the complex correlation structure. We assessed the multilevel model by comparing with the linear mixed-effects (LME) models using "dose" genotypes on chromosome 3 and the 200 simulation replicates of longitudinal response and covariates provide by Genetic Analysis Workshop 18 (GAW18) [8].

## Methods

### Method 1: LME model

Linear mixed-effects models offer a natural approach to deal with correlation structures among observations. For longitudinal family data, we can define an LME model:

(1) $$y_{ijk}=x_{ijk^{\prime }}\beta +z_{ijk^{\prime }}\gamma_{k}+\epsilon_{ijk},$$

where $y_{ijk}$ is response of the *i*th repeated measure of the *j*th individual in the *k*th family, where $i=1,\ldots ,n_{jk}$, $j=1,\ldots ,m_{k}$, and k=1,…,K, with $n_{jk}$ being the number of measures for individual *j *in family *k *and $m_{k}$ being the number of individuals in family *k*; $x_{ijk}$ is a covariate vector (including genotype) for fixed effects *β*. $z_{ijk}$ is a covariate vector for random effects $\gamma_{k}$, where $\gamma_{k}:={\left(\gamma_{1k}\ldots \gamma_{m_{k}k}\right)}^{\prime }\sim N\left(0,D_{k}\right)$, $D_{k}$ the covariance matrix among individuals in family *k *(e.g., the kinship matrix). Also, $\epsilon_{jk}:=(\epsilon_{1jk}\ldots \epsilon_{n_{jk}jk})\sim N(0,{\text{Σ}}_{jk}),$ where ${\text{Σ}}_{jk}$ is the covariance matrix among the repeated measures for individual *j *in family *k*. We assume $\gamma_{k}$ and $\epsilon_{jk}$ are independent between each other and among themselves for all *j *and *k*. To implement the LME model, we applied the following R package:

**GWAF: **R package GWAF was design for genome-wide analysis for family data [9]. It accounts for the pedigree correlation structure by kinship matrix. However, it does not handle longitudinal repeated measures. So this method was used to represent the cross-sectional analysis for family data and was compared with other family-data analysis incorporating longitudinal information.

**Lmekin: **R function lmekin in package coxme [10] was applied to account for both the family correlation structure and the correlation structure of the longitudinal repeated measures. Specifically, we set the model that includes a random intercept at individual level to account for the correlation of repeated measures assuming compound symmetry structure, a random intercept at family level to account for the clustering effect among family members. Furthermore, the kinship matrix was incorporated through its varlist option to account for the kinship correlation among family members.

### Method 2: Multi-level model

We extend the classic multi-level model [4,5,11] to analyze WGS family data with longitudinal repeated measures. The response for the *i*th measure (level 1) of the *j*th individual (level 2) in the *k*th family (level 3) can be written as

(2) $$y_{ijk}={x_{ijk}}^{\prime }\beta +u_{k}+g_{jk}+v_{ij}+e_{ijk},$$

where ${x_{ijk}}^{\prime }$ and *β *are similarly defined in (1). The rest random-effect terms on the right side of the equation are normal distributed with mean zero and variance characterizing the correlation structure among observations. Denote the response vector $y=\left(y_{ijk}\right)$. We have y~N(xβ,V), where

(3) $$Var\left(y\right)=V=A\sigma_{u}^{2}+B\sigma_{g}^{2}+C\sigma_{v}^{2}+I\sigma_{e}^{2}.$$

The first random term $u_{k}$ characterizes the clustering effects at the family and individual levels. Specifically, $A={⊕}_{k}\left(J_{k}⊗J^{*}\right)$, where $J_{k}$ is a matrix of 1's with dimension being the size of *k*th family, $J^{*}$ is a matrix of 1's with dimension being the number of repeated measures per individual. ⊕ denotes the matrix direct sum, and ⊗ denotes the Kronecker product. The second random term $g_{jk}$ indicates the genetic correlation (kinship coefficients) among individuals in the *k*th family. Mathematically, $B={⊕}_{k}\left(D_{k}⊗J^{*}\right)$, where $D_{k}$ is the kinship matrix. The third random term $v_{ij}$ indicates the correlation among repeated measures in the *j*th individual: $C={⊕}_{k}\left(I_{k}⊗R\right)$, where $I_{k}$ is an identity matrix with dimension being the size of the *k*th family and, *R *is the correlation matrix among repeated individuals. For example, if we assume compound symmetry structure, for three repeated measures,

(4) $$R=\left(\begin{array}{ccc} 1 & \rho & \rho \\ \rho & 1 & \rho \\ \rho & \rho & 1 \end{array}\right)=\left(\begin{array}{ccc} 1 & 0 & 0\\ 0 & 1 & 0\\ 0 & 0 & 1 \end{array}\right)+\left(\begin{array}{ccc} 0 & 1 & 1\\ 1 & 0 & 1\\ 1 & 1 & 0 \end{array}\right)\rho .$$

So the term can be decomposed as $C\sigma_{v}^{2}=C_{1}\sigma_{v}^{2}+C_{2}\rho \sigma_{v}^{2}$, such that the matrixes are all known and the parameters can be estimated as described below. Certainly, more complicated correlation structure can be modeled by a further decomposition according to the number of covariance parameters to be estimated. Finally, $e_{ijk}$ is the independent and identically distributed error term, and *I *is the identity matrix for all observations.

For the inference of the multilevel model, the IGLS algorithm [6,7] is applied. Let ỹ=y-Xβ. Note that

(5) $$E\left(\tilde{y}{\tilde{y}}^{\prime }\right)=V=A\sigma_{u}^{2}+B\sigma_{g}^{2}+C_{1}\sigma_{v}^{2}+C_{2}\rho \sigma_{v}^{2}+I\sigma_{e}^{2}.$$

Step 1: Given *β*, estimate *V *by the least squares estimation of variance [12]. Specifically, this is a procedure of fitting regression model of response vector $y^{*}=vec\left(\tilde{y}{\tilde{y}}^{\prime }\right)$ to the design matrix $X^{*}=\left[vec\left(A\right),vec\left(B\right),vec\left(C_{1}\right),vec\left(C_{2}\right),vec\left(I\right)\right]$, where $vec\left(A\right)$ denotes the vectorization of the upper triangular part of matrix *A*. So,

(6) $${\left({\hat{\sigma }}_{u}^{2},{\hat{\sigma }}_{g}^{2},{\hat{\sigma }}_{v}^{2},\hat{\rho \sigma_{v}^{2}},{\hat{\sigma }}_{e}^{2}\right)}^{\prime }={\left(X^{{*}^{\prime }}\left(V^{-1}⊗V^{-1}\right)X^{*}\right)}^{-1}X^{{*}^{\prime }}\left(V^{-1}⊗V^{-1}\right)y^{*}\;\text{and}\;\hat{\rho }=\hat{\rho \sigma_{v}^{2}}/{\hat{\sigma }}_{v}^{2}$$

Step 2: Given *V*, estimate *β *by the weighed least squares estimate:

(7) $$\hat{\beta }={\left(x^{\prime }V^{-1}x\right)}^{-1}x^{\prime }V^{-1}y.$$

The estimation procedure starts at an arbitrary *β *(e.g., obtained from a multiple regression fitting) and then iterates between steps 1 and 2 until convergence. Because the IGLS estimate is equivalent to the restricted maximal likelihood estimate [4], we can apply a Z-test to calculate *p-*values for the elements in $\hat{\beta }$, which contains the fixed genetic effects. In particular, because $Var\left(\hat{\beta }\right)={\left(x^{\prime }V^{-1}x\right)}^{-1}$, the Z-test statistic for $\beta_{j}$ is $Z_{j}={\hat{\beta }}_{j}/Var{\left(\hat{\beta }\right)}_{jj}$, and the two-tailed *p-*value is $p_{j}=\text{Pr}\left(\left|N\left(0,1\right)\right|>\left|Z_{j}\right|\right)$. Certainly, this multilevel model has the potential to be further extended to incorporate a more complicated covariance structure for more sophisticated modeling.

## Results

For evaluating the methods, we used the "dose" genotype data of the 169 true single-nucleotide variants (SNVs) on chromosome 3 that were associated with diastolic blood pressure (DBP) in 200 simulation replicates. These data contain 849 individuals in 20 families, and the number of individuals in families is 21 to 74, with the mean 42.45 and the median 36.5. Kinship matrixes of these families were directly calculated based on the pedigree information. The above models were fitted with or without covariates: age, blood pressure medicine status, and sex. For GWAF, which does not analyze longitudinal data, we applied the DBP at the first time point as the response. For lmekin and multilevel model, we applied all three longitudinal repeated measures. The knowledge of the true SNVs was only used for evaluating the power of these association tests, not for the data analysis strategy.

**Figure 1:**
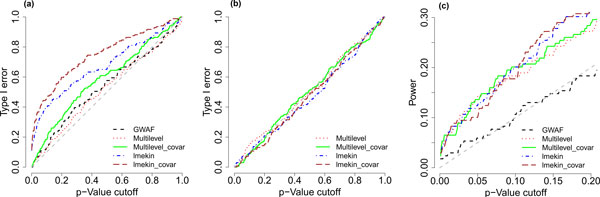
**Type I error and power for detecting DBP-related**. single-nucleotide variants (SNVs) on chromosome 3. Considering all 169 diastolic blood pressure (DBP)-related SNVs on chromosome 3, the type I error rates were estimated by the false positive rates when Q1 was the null response (a) and when the genotypes are permuted (b); the power was estimated by the true positive rate when DBP was the response (c). A model with or without containing covariates (age, blood pressure medicine status, and sex) is denoted by its name with or without "covar".

First, we evaluated the type I error rate control for these methods. Fitting the 169 DBP-related SNVs on chromosome 3 to Q1, a null response provided by GAW18 "to facilitate assessment of type I error," we plotted in Figure 1a the false-positive rates over a variety of *p-*value cutoffs. It is clear that the type I error rate of lmekin is highly inflated, and the type I error rates of multilevel model and GWAF are closer to the expected level around the diagonal line. The inflation is worse when covariates are contained in the models (denoted "covar"). We also studied the type I error rate through permutation. Figure 1b shows the false-positive rates for fitting the permuted genotype data of these SNVs to DBP response, which retained the relationship between covariates and DBP but destroyed the association between SNVs and DBP. Now both lmekin and our multilevel models control the type I error rate perfectly well. To explain the puzzle, we checked the GAW18 "answers" and found that Q1 was simulated as a quantitative trait correlated among family members with heritability 0.68, but the total heritability for DBP is only 0.317. This means that Q1 values have stronger correlation than DBP values do. The inflation of the type I error of lmekin indicates that this LME model is less capable than our multilevel model in accounting for the correlation among individuals (cf. [13]).

We studied the power of detecting the 169 DBP-related SNVs on chromosome 3. Based on the phenotype data in the simulation replicate 1, Figure 1c shows the true positive rate of detecting these true SNVs over a variety of *p-*value cutoffs. In general, the power of detecting true SNVs is low at small or moderate *p-*values. This phenomenon indicates that the sample size is still relatively too small to detect a large proportion of the weak genetic effects simulated in the data. At the same time, longitudinal methods (lmekin and multilevel models) are better than the one-time-point model (GWAF); the latter does not have much power except for the strongest SNVs. The lmekin and the multilevel models have similar performance overall, but the multilevel model is better at the region of relatively small *p-*values (e.g., *p-*value <0.1) that are of practical interest. For both lmekin and multilevel models, there is no big difference between the models with and without covariates. We also studied the power of detecting specific SNVs by using the data of 200 simulation replicates. For example, by the multilevel model with covariates, the strongest SNV at location 48040283 always got significant *p-*values from 1.8 × 10^−31 ^to 3.09 × 10^−9^.

## Discussion

In this work, our main focus was to see whether modeling longitudinal data could provide helpful information to increase the power of detecting true SNVs when compared with the methods for analyzing data at one time point. Here we directly applied the original genotype data into modeling and illustrated that the longitudinal repeated observations were indeed helpful to detect DBP-related genetic factors. However, many true SNVs are rare variants, some of which could have big allelic effect for specific individuals when the disease mutation presents. As a result of small minor allele frequency (MAF), the association between such rare variants and their corresponding phenotypes is still weak at the population level [1]. This may be one of the main reasons why the overall power is low in detecting the majority of the causal or regulatory genetic factors. Various strategies of rare-variant collapsing procedures [14,15] could be applied to grouping and combining genotypes of rare variants, which has potential to further increase the power.

The computational speed of the multilevel model is comparable with the linear mixed-effects model estimation by lmekin. Both models are computationally demanding (e.g., ~10 minutes for our implementation of multilevel model and 8 minutes for lmekin to process one SNV on a MacBook Pro with 2.9-GHz Intel Core i7). However, we observed that the convergence speed of the iterative generalized least squares algorithm for the multilevel model is relatively fast: the results usually do not change much after two iterations. So, restricting the number of iterations could potentially reduce computational time. Further study on improving computation efficiency will be carried out in the near future.

## Conclusions

We developed a multilevel model for fitting family-based genotype data and repeated measures of covariates to quantitative longitudinal response, which accounts for correlations among individuals, nesting effects at the family and individual levels, and the time series correlations due to the repeated measures of covariates and responses. Using the simulated data of GAW18, this method showed more accurate type I error control than the LME model by lmekin, which is likely the result of better account for correlations among individuals. The multilevel model also provided higher power at small *p-*value cutoffs. At the same time, both lmekin and multilevel model, which use the longitudinal information, have higher power than GWAF, which only models data at one time point.

## Competing interests

The authors declare that they have no competing interests.

## Authors' contributions

ZW designed the overall study. ZW, TC, and PS conducted statistical analyses and drafted the manuscript. All authors read and approved the final manuscript.
